# Prevention of Sepsis in Nasal Operations

**Published:** 1904-05-28

**Authors:** 


### PREVENTION OF SEPSIS IN NASAL OPERATIONS.

Although sepsis following nasal operations is ordinarily of a mild type, there is not sufficient reason to neglect antiseptic precautions altogether, for occasionally severe consequences follow, such as infection of the accessory sinuses and of the middle ear, or even meningitis. Even if no severe infection takes place, the observance of antiseptic principles is likely to be followed by quicker and more satisfactory recovery. Dr. H. Beaman Douglas[1] recommends that before every nasal operation the skin of the nose and face should be carefully cleansed and disinfected. If there be a moustache it must be removed if any cutting of the integument of the nose is intended, in other cases it will be sufficient to treat it with soap and water and antiseptics, and to exclude it from the field of operation by a piece of gauze fastened in position with strapping. Whether the moustache is removed or not, the hairs in the vestibule of the nose must be clipped away. Three hours before the operation the interior of the nose should be washed, first with a solution of hydrogen peroxide (10 vol. strength). This may be done with a spray. The interior of the nose should then be douched with weak saline solution (a teaspoonful of salt to a quart of water); or if there be much pus or mucus in the nose a solution of 10 drops of tincture of green soap in a quart of water should be used. Whatever solution be employed for the douche it is important to have it warm (102° to 106° F.), and to inject it slowly and very gently, and in a backward direction, parallel with the floor of the nasal cavity, and with the patient's head bent well forward over a basin. If these precautions are not observed, and the liquid is injected forcibly, or too hot or too cold, not only may it cause much pain and discomfort, but there will be an actual danger of causing damage to the middle ear or accessory sinuses.

[1] Post-Graduate, May.

